# Does fast running limit numerical variability of the vertebral column in rabbits and hares (Leporidae: Lagomorpha)?

**DOI:** 10.1098/rsos.241813

**Published:** 2025-01-29

**Authors:** Megu Gunji, Nuttakorn Taewcharoen, Fumio Yamada, Emma Sherratt

**Affiliations:** ^1^ Department of Life Sciences, Faculty of Life Sciences, Toyo University, Saitama 351-0007, Japan; ^2^ School of Biological Sciences, Faculty of Sciences, Engineering and Technology, University of Adelaide, Adelaide, South Australia 5005, Australia; ^3^ Okinawa University, Kokuba 555, Naha, Okinawa 902-0075, Japan; ^4^ Amami Rabbit Museum QuruGuru, Mahoroba Park, Yamato, Kagoshima 894-3104, Japan

**Keywords:** intraspecific variation, transitional vertebra, anomaly, performance

## Abstract

In mammalian vertebral columns, locomotive ability is expected to be an evolutionary driver of variation in the number of vertebrae; in species evolved to run fast or have a flexible vertebral column, they generally have limited numerical variation and low occurrence of malformed vertebrae to maintain their running performance. Although this hypothesis is supported among species sharing similar locomotive constraints (e.g. dorsomobile versus dorsostable species), whether it applies at the within-species level is unknown. We test this hypothesis using species of Leporidae (rabbits and hares) with different locomotive abilities: we examined the number of presacral vertebrae and the frequency of abnormalities in 504 specimens from 4 species, representing cursorial, saltatorial and generalist modes. Our results show that the cursorial leporids had the lowest numerical variability and fewest abnormalities within species, although this was not statistically different from saltatorial or generalist species. We also identified 11 conditions of vertebral abnormality previously unexplored and theorize that each may pose different degrees of locomotive impairment and effects on species’ fitness. The lack of statistical support for the hypothesis at a finer phylogenetic level suggests further research is needed to understand whether numerical variability is under stabilizing selection or a developmental response to locomotive constraints in cursorial animals.

## 1. Introduction

Vertebral numbers have long been recognized to vary greatly among vertebrate species [1–4], and such findings continue to shed light on the evolution of the vertebrate body plan. At a developmental level, numerical variation is due to two levels of genetic control: the number of somites for the number of vertebrae (by clock-and-wavefront mechanism [5]), and the developmental fate of somites for vertebral column segmentation (by *Hox* genes [6]) [7]. The regulation of how somites are formed (somitogenesis) is conserved across vertebrates [8], yet each lineage has its own developmental ‘clock’ and *Hox* expression pattern. This leads to not only a variation in the total number of vertebrae [7] but also the variation in the number of vertebrae in each region [9,10]. These two processes of developmental regulation result in numerical variation, which manifests among individuals (within species) and among species. At the within-species level, the variation is known to occur in response to ecological pressures, such as temperature in fish [11]. For the among-species level, in addition to the pressure from ecological factors, such as precipitation in salamanders [12] or locomotion in snakes [13], the phylogeny also has a strong role [14]. The mammalian vertebral column, despite being phylogenetically conserved in the total number of vertebrae (excluding the tail), has well-defined vertebral column regions [15], with the numerical variation across regions distinctly varied among species [14].

Regionalization of the vertebral column is hypothesized to optimize different functional roles performed by the backbone [16]. Five morphologically differentiated vertebral groups can be classified: cervical, thoracic, lumbar, sacral and caudal [9]. As the pre-sacral region (cervical, thoracic and lumbar) provides dominant support to the body during locomotion [17], the number of vertebrae in each region varies greatly across different modes of mammalian locomotion [14]. Large numerical variability persists still even when narrowing down to the variation within terrestrial mammals (e.g. [4,18–21]). The variability in vertebral number will directly influence the flexibility and stability of the column [12,22,23]. There is a strong association between numerical variability and running adaptation among land mammals—broadly speaking, the species having a flexible back (dorsomobile) or specialized in running (cursorial) commonly show a lesser degree of variability than the species with a stiffer back (dorsostable) or not so much of a running specialist [20,21].

In addition to the variation in vertebral number within regions, there is a variation due to congenital vertebral anomaly, which can be either symptomatic or non-symptomatic [24]. Vertebral anomalies have been documented in both domestic (e.g. [24–27]) and wild animals (e.g. [28,29]). The commonest type of anomaly is a transitional vertebra (TV)—a condition where a vertebra at the boundary between adjacent regions shows morphological characters from both regions [30]. The TV is found in several mammalian species (e.g. [25,27,31,32]), including humans (e.g. [33,34]). The development of TV is caused by the partially mutated *Hox* genes, leading to an incomplete homeotic transformation of a vertebra at the boundary between two regions. The presence of TV can impair vertebral flexibility, but the extent to which it can pose is variable [24,27]. So far, one type of TV (the lumbosacral TV, LSTV) has been examined for the relationship between its occurrence and running abilities [20]. Yet, there are also cervicothoracic TV (CTTV) and thoracolumbar TV (TLTV) [35].

Since the number of vertebrae and the presence of malformed vertebra can have direct impacts on vertebral column flexibility [11,36], it is expected that limiting variation of these features better maintains optimal flexibility and stability, thus maintaining the locomotive performance. This expectation is supported by an analysis focusing on broad-scale patterns across the mammalian order, where species sharing similar locomotive pressure show a similar degree of numerical variability [20,21]. However, since the number of vertebrae is associated with phylogeny [14], it is still unknown whether this relationship applies at a finer scale of phylogenetic level, particularly within species. By examining species that are closely related, the influence of phylogeny can be minimized [37]. To provide more evidence towards this relationship, we choose the family Leporidae as a study system.

Leporidae is a mammalian family comprising around 75 species of hares and rabbits [38]. They inhabit all terrestrial habitats (except Antarctica), leading to a diversity in their locomotive ability [38–40]. This ranges from highly cursorial species specialized in running (*Lepus* spp.) to species that are saltatorial/hopping (*Oryctolagus*) and generalist species moving by slow bounding or scampering (*Pentalagus*) [41,42]. These modes are reflected in trait diversity, such as skull tilting angle [43] and limb proportions [44,45]. Their vertebral columns have been found to show some degree of morphological specialization for running, convergent with canid predators [46]. Ultimately, these characteristics make Leporidae an ideal system to examine the relationship between vertebral number variability and running adaptation.

To our knowledge, comparative studies of vertebral variation within Leporidae are overlooked in research; the most complete thus far being Taewcharoen *et al*. [46], while the focus is mostly on the domesticated European rabbit [27,35,47–50] or the captive hares [51]. Among broad-scale comparisons of mammals, a few leporid representatives have been included [14,21,52] but omitted from others [20,53]. In addition, the number of individuals observed is usually limited (<20 individuals per species), which is likely to be a common constraint in many research studies aiming to study a sufficiently complete vertebral column (e.g. [14,19,54]). This leaves a knowledge gap on the within-species variability.

In this article, we aim to test whether the relationship between variation in pre-sacral vertebral number (including anomalies, i.e. TV) and running ability holds for Leporidae by focusing on differences among species in their within-species variation. We overcome the limitation of a low number of individuals with complete skeletal specimens by observing more than 500 individuals across four leporid species covering all three levels of locomotory gradient in Leporidae. We hypothesize that the highly cursorial species will show the lowest degree of vertebral variation, in terms of variance in number and instances of anomalies, and the opposite for less cursorial ones.

## 2. Material and methods

### 2.1. Specimen acquisition

**Figure 1.**
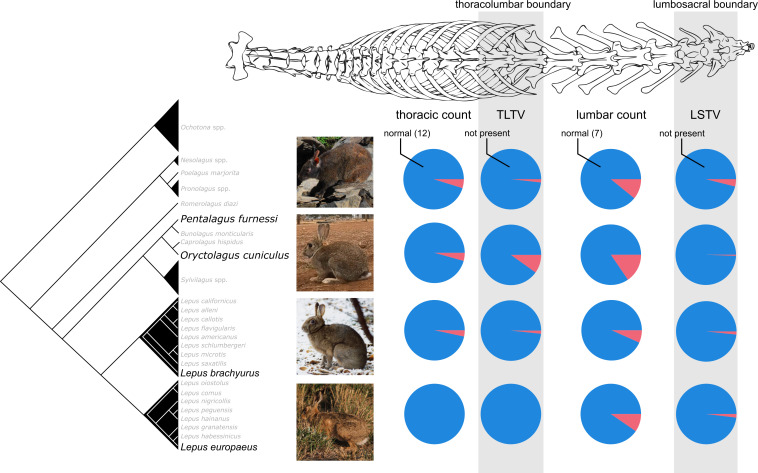
Phylogenetic relationship of the four leporid species studied herein and their respective proportions of vertebral anomaly (red pie slice) in each vertebral region. The designation ‘normal’ refers to the normal number of vertebrae in the thoracic (12 vertebrae) and lumbar (7 vertebrae) regions; and ‘not present’ refers to no transitional vertebra in the boundary region. N.B.: The phylogeny is simplified from Iraçabal *et al*. [55] and Matthee *et al*. [56]; monophyletic clades are represented by triangles with different sizes reflecting species diversity; sampled *Lepus* species are from two clades. Abbreviations: TLTV = thoracolumbar transitional vertebra; LSTV = lumbosacral transitional vertebra. Images courtesy of Hiromitsu Katsu (*P. furnessi*), Nuttakorn Taewcharoen (*O. cuniculus*), Yasuko Segawa and Fumio Yamada (*L. brachyurus*) and Philip Stott (*L. europaeus*). The image of the vertebral column is for illustrative purposes.

Vertebral specimens from wild populations of four leporid species were studied: *Lepus europaeus*, *Lepus brachyurus*, *Oryctolagus cuniculus* and *Pentalagus furnessi*. These represent the three modes of locomotion (cursor, saltator and generalist) in Leporidae and distantly related [55,56] (figure 1). Sex was not identified for most museum specimens, however, the sexual dimorphism in numerical variation of pre-sacral vertebrae is negligible (but see [47]). Vertebral columns of *L. europaeus* and *O. cuniculus* were scanned using medical computed tomography (CT) scanners: by X-ray (SOMATOM Force, Siemens Inc.) and by photon counting (NAEOTOM Alpha, Siemens Inc.). The use of two scanners was due to an upgrade of the infrastructure during the study. The resulting image stacks were used to reconstruct digital three-dimensional models using three-dimensional Slicer v. 5.4.0 [57,58], and the three-dimensional models were used to examine the vertebral columns of these two species. For *L. brachyurus* and *P. furnessi*, skeletal specimens stored in multiple Japanese museums were used. Table 1 shows the number of individuals examined for each species and their respective collection, with the complete list of specimen catalogue numbers provided in electronic supplementary material, table S1. Specimens of the Laboratory of Dr. Sherratt at the University of Adelaide were obtained from pest control across Australia (ethics permit: ORA 19/22/020, CA 2019/06/1288, AEC 18/5/12) and from the concurrent study by NT (ethics permit: S-2022-038) (digital specimens available on morphosource.org under project ID 000671799). For *P. furnessi*, the specimens housed at the National Museum of Nature and Science, Tokyo were collected and prepared from carcasses found in the wild by the Amami Wildlife Conservation Centre of the Ministry of the Environment, Government of Japan. For all *L. brachyurus* specimens and *P. furnessi* specimens that are housed in other museums, no detailed records are available on how the specimens were collected. We included both adult and juvenile specimens in our dataset. Although the juvenile specimens do not have their vertebral epiphyseal plate fused to the centrum [59], vertebral processes are fully ossified and presented on the skeletal specimens, and the malformation could be observed as early as in newborn animals [47].

**Table 1.** Species observed in this study.

species	number of individuals	modes of locomotion
*Lepus europaeus*	52	cursorial
*Lepus brachyurus*	60	cursorial
*Oryctolagus cuniculus*	162	saltatorial
*Pentalagus furnessi*	230	generalist

### 2.2. Vertebrae identification

**Figure 2.**
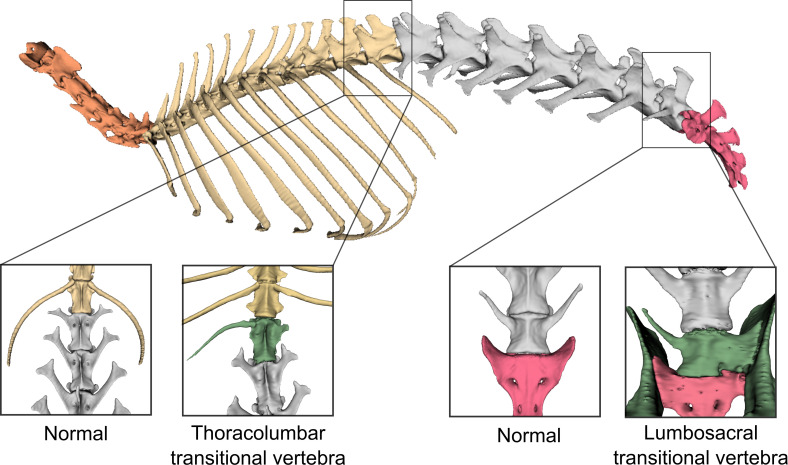
Digital three-dimensional models of the leporid vertebral column (represented by a normal *L. europaeus*), with different colours identifying each vertebral type in normal condition: cervical (orange), thoracic (yellow), lumbar (grey) and sacral (pink) vertebrae. The inset indicates a location where a transitional vertebra (green) is present. For lumbosacral transitional vertebra, an ilium is also shown in green colour. Cervicothoracic transitional vertebra is not observed, thus not shown. Image not to scale.

We identified vertebrae using conventional vertebral regionalization in mammals: cervical vertebrae comprise the atlas, axis and subsequent vertebrae bearing no movable rib articulation; thoracic vertebrae show costal fovea on their centrum for movable ribs, except the last pair of ribs which may be rudimentary or become ankylosed (immovable); lumbar vertebrae are free from rib and have transverse processes that do not form sacral foramina with the following vertebra; and sacral vertebrae are composite vertebrae with the lateral portion between two adjacent vertebrae forming sacral foramina [60–62] (figure 2). Caudal vertebrae were not considered in this study. A TV is defined as a vertebra having characters of two adjacent regions unilaterally on each side, i.e. CTTV, TLTV and LSTV. We treated a TV as its own type of vertebra rather than considering it as a numerical half between two adjacent regions. In the case of a vertebra at the thoracolumbar boundary with bilaterally proper transverse processes and a pair of rudiment ribs that do not articulate with the vertebral body, we classified it as a lumbar vertebra.

We counted the number of vertebrae in each region. The combination of numbers is present as a vertebral formula in the form: # cervical/# thoracic/# lumbar. When the TV was observed, we designated a separate number in the formula following the order in which it was presented. The formula with the highest frequency across all species was considered the normal vertebral formula. Any deviation from the normal formula was considered abnormal. We recognized there are other types of congenital vertebral anomalies [24]. However, we did not observe them in our collection and their pathology is not discussed here. Throughout this article, we preserve the term ‘anomaly’ only when referring to the TV.

### 2.3. Statistical analysis

All analyses were done in the R statistical environment v. 4.4.1 [63]. Calculations were done using functions in base R and the *stats* package v. 4.4.1 [63].

The vertebral formula was recorded for each species. Prior to statistical analysis, we simplified the frequency of all observed vertebral formulae down to the frequency of normal formula and abnormal formula. To test whether the normal–abnormal frequency was associated with species, Fisher’s exact test was performed using the contingency table (2 × 4 table). Pairwise Fisher’s exact test was also calculated between each pair of species (2 × 2 sub-table), with the *p*-value corrected using the Holm method following Galis *et al*. [20]. We also calculated the ratio between the abnormal and normal formulae for each species. The 95% confidence interval of each species’ ratio was calculated by a bootstrapping approach to represent a scenario in which all species were examined from the same number of individuals. The bootstrapped ratios were calculated from 40 individuals subsampled from each species without replacement for 9999 iterations. We chose 40 individuals as this was 75% of the species with the smallest sample size (*n* = 52 in *L. europaeus*). The R function set.seed() at the arbitrary value ‘3’ was used to ensure the reproducible subsample.

## 3. Results

A total of 12 vertebral formulae was observed, with the most frequent formula (and hereafter referred to as ‘normal’) being 7/12/7 (cervical/thoracic/lumbar), without any presence of TV. The occurrence of the normal formula was 82.7–91.7% across the four leporid species (table 2). The remaining 11 formulae (hereafter ‘abnormal’) could be classified into three groups—single-, double- and triple-deviation—following the instances of deviation from the normal formula. The single-deviation group comprised five conditions: one extra thoracic, one extra lumbar, one absent thoracic, one absent lumbar and one LSTV. For the double-deviation group, there were five conditions: one extra thoracic with one lumbar absent, one thoracic absent with one extra lumbar, one thoracic absent with one LSTV, one TLTV with one lumbar absent and one lumbar absent with one LSTV. And for the triple-deviation group, there was only a sole condition of one extra thoracic with one lumbar absent with one LSTV (table 2). When focusing on the presence of TV in these 11 abnormal formulae, one formula involved TLTV and four involved LSTV. No CTTV was found.

**Table 2.** Frequency of each vertebral formula (cervical/thoracic/lumbar) observed in four leporid species, presented in order from most to least athletic, left to right. The ratio between abnormal and normal formulae presented with a 95% confidence interval from bootstrapping of 40 resampling individuals without replacement. Abbreviation: TLTV = thoracolumbar transitional vertebrae; LSTV = lumbosacral transitional vertebrae.

grouping	formula	*Lepus europaeus*	*Lepus brachyurus*	*Oryctolagus cuniculus*	*Pentalagus furnessi*
total		52	60	162	230
normal	7/12/7	47	55	134	197
single vertebra anomaly					
single extra thoracic	7/13/7			2	
single extra lumbar	7/12/8		1	2	1
single absent thoracic	7/11/7		1		4
single absent lumbar	7/12/6	4		2	10
single lumbosacral transitional	7/12/7+1			1	3
double vertebrae anomaly					
single extra thoracic + single absent lumbar	7/13/6		1	5	4
single absent thoracic + single extra lumbar	7/11/8				1
single absent thoracic + lumbosacral transitional	7/11/7+1				1
single absent lumbar + thoracolumbar transitional	7/12+1/6		1	16	4
single absent lumbar + lumbosacral transitional	7/12/6+1	1			4
triple vertebrae anomaly					
single extra thoracic + single absent lumbar + lumbosacral transitional	7/13/6+1		1		1
total individual with TLTV			1	16	4
total individuals with LSTV		1	1	1	9
abnormal:normal ratio		0.106	0.091	0.209	0.168
95% confidence interval		0.053–0.143	0.026–0.143	0.081–0.379	0.053–0.333

The frequency of each formula varied from species to species. In *L. europaeus*, two abnormal formulae were found, while in *L. brachyurus*, five abnormal formulae were observed. TLTV was found in *L. brachyurus* but not in *L. europaeus*; LSTV occurred in both species. In *O. cuniculus*, six abnormal formulae were found—one formula involved TLTV, and one involved LSTV. And in *P. furnessi*, all abnormal formulae were recorded, except one formula—a single extra thoracic. The species with the highest occurrence of TLTV was *O. cuniculus*, while of LSTV was *P. furnessi* (table 2).

For the ratio between abnormal and normal formulae, *L. brachyurus* showed the lowest rate of abnormality (9.1%), followed by *L. europaeus* (10.6%), *P. furnessi* (16.8%) and *O. cuniculus* (20.9%) (table 2). The bootstrapped 95% confidence interval from 40 individuals subsampled had an overlapping range across all four species, indicating the non-significant differences across the observed ratios. Similarly, Fisher’s exact test showed no significant relationship between species and abnormality in either overall count or pairwise comparison (adjusted *p*‐value > 0.05).

## 4. Discussion

Adaptation towards fast running is hypothesized to limit evolutionary change in the vertebral column, particularly reducing individual variation in the number of vertebrae in each region and the occurrence of abnormalities in highly cursorial animals [20]. We tested this hypothesis at a finer phylogenetic scale, with large within-species samples. Our results from four species trended towards this pattern, but were not statistically significant: the two fast-running cursorial leporids (*Lepus* spp.) showed less within-species numerical variability than saltators and generalist bounders (*O. cuniculus* and *P. furnessi*, respectively). Variability in the saltatorial *O. cuniculus* was higher than in the generalist *P. furnessi*, which was unexpected. Instead of numerical variance, in leporids, the type of abnormality may be more relevant when considering locomotive ability. We found that the frequency of abnormal formulae did not equally occur in each species: TV were almost absent from the cursorial leporids (3 out of 112 individuals of *Lepus* had a TV). Again, these differences were not significant. However, we identified 11 distinct conditions of abnormality, which may pose different degrees of locomotive impairment, resulting in different individual fitness and trait inheritance. We discuss whether the important factor is speed or mobility acting on variance, whether abnormalities are more important than numerical variation, and if population-level genetic effects may influence these results.

### 4.1. A trend, not statistically supported

The rate of abnormal vertebral formulae we observed was 9.1–10.6% for the cursorial leporids, and 16.8–20.9% for the saltatorial and scampering leporids (table 2). The difference in our sample was not as wide as reported by Galis *et al*. [20], with 0.4–5% in fast-running mammals and 25–70% in slower runners. This is surprising given the broad range of maximum speeds in Leporidae [45] with *L. europaeus* being an extreme athlete achieving 72 km h^−1^ [64]. Using a larger taxonomic range, Williams *et al*. [21] argued that the running speed did not strongly explain the numerical variance of the taxa; rather, the broader categories, such as the mobility of the column (dorsostable versus dorsomobile species) or the broad locomotive modes (running versus climbing), were more associated with the variance. In anthropoids (monkeys, apes and humans), vertebral formulae are highly variable within species [18,65] with only humans and eastern gorillas presenting significantly lower variance due to their highly distinct locomotion and habitats [65]. Although not specifically mentioned, Williams and colleagues’ results implied that the variance in vertebral formulae did not differ across other anthropoid species. Therefore, further work is needed to examine the vertebral column mobility in Leporidae, because our results imply that these four leporid species might have a more similar column mobility than expected given their running ability. Behavioural differences of these species are further discussed below. We encourage more studies among closely related species testing this hypothesis to better understand the factors responsible.

### 4.2. Vertebral abnormality, locomotion and fitness of wild leporid populations

Differences in the running abilities of leporids have left adaptive signals in their skeleton, such as the skull tilting angle [43], limb proportions [44,45] and potentially the shape of the vertebral column as well [46]. Behaviourally, these differences are reflected in species’ escape strategies, which are most often contrasted between hares (herein represented by *Lepus* spp.) and rabbits (herein represented by *O. cuniculus* and *P. furnessi*). The hares adopt a sit-and-wait strategy, where they will remain silent until the disturbance (e.g. predators or approaching humans) gets perilously close to the hares, and the hares will sprint and try to outrun the approacher ([41], N Taewcharoen, personal observation). In contrast, the rabbits will notice the disturbance from afar and escape to their shelter immediately rather than remaining motionless or trying to outrun the approacher ([41], N Taewcharoen, personal observation). These behaviours are also reflected by the habitat preference of hares and rabbits: the hares live in large and open areas where they can run very quickly without any obstacles, while the rabbits prefer areas with abundant scrubs (in which they can hide) or with loose substrate (through which they can dig their shelter) ([66,67], N Taewcharoen, personal observation). The extreme case is for *P. furnessi* (an Amami rabbit; the least cursor herein) whose habitat is a dense forest or characterized by being steep and rocky [68,69]. Their escape strategy is to move back to their not-well-hidden burrows, which are within 100–200 m from their feeding ground [68,70]. Given these behavioural differences, we expect they would have different tolerance levels to the locomotive impairment posed by vertebral abnormality, particularly by the TV.

For quadrupedal mammalian runners, the sagittal flexibility (dorsoventral motion) in the lumbar region is essential [71], in which the lumbosacral intervertebral joint contributes the most compared with other lumbar intervertebral joints [72,73]. The LSTV is one of the vertebral anomalies known to be associated with degenerative lumbosacral joint, thus limiting dorsoventral flexibility [20,74–76]. This poses a negative impact on the performance of locomotion, ultimately reducing the survival rate of the animal [28]. In an agile species, whose mobility of the vertebral column is vital [71], the impact of compromised flexibility by the LSTV is expected to be more pronounced than in less agile species. Consequently, the selective pressure against the presence of LSTV in agile species should be high. This is likely to explain the single observation of LSTV in leporids with a certain degree of running capability (*Lepus* spp. and *O. cuniculus*), but relatively higher, though non-significant, in *P. furnessi,* which do not really run.

The other type of anomaly found in this study, the TLTV, showed the highest frequency in *O. cuniculus*. Functionally, the TLTV have been less studied, and the extent to which they affect the column is under debate even in humans [24,77,78]. In a concurrent study, one author (NT) quantified the running behaviours of two rabbits with TLTV (formula 7/12 + 1/6); their running performance was not evidently different from the rabbits with a normal vertebral formula (N Taewcharoen, personal observation). The gross dissection of the paravertebral musculature of those rabbits showed that the muscular arrangement for the longissimus muscle onto the TLTV was the same as with the preceding thoracic vertebrae. For thoracic iliocostal and quadratus lumborum muscles, their origins and insertions on TLTV in abnormal individuals were weaker than on the normal last thoracic vertebra in normal individuals (N Taewcharoen, personal observation). Since the locomotive function of the thoracic iliocostal muscle is to stabilize the column in lateral motion, and of the quadratus lumborum muscle to either flex or stabilize the column in dorsoventral motion [79–81], their weak attachments on the single TV may not significantly compromise the function of the whole muscles. In domestic pigs, the column curvatures in the thoracolumbar boundary with TLTV appeared to be normal, despite the whole back being diagnosed as ‘dipped back condition’ (spinal lordosis) [82]. And in domestic small mammals, including rabbits, the presence of TLTV is generally asymptomatic [24,35]. Altogether, this suggests that the presence of a TLTV does not directly impair the rate of survival in wild species, under a certain environmental pressure. And when more individuals with an anomaly survive to adulthood, the probability of giving birth to offspring with the anomaly becomes high [47].

We also observed the trend towards losing rather than gaining a lumbar vertebra. When a TV presented (either TLTV or LSTV) or when supernumerary thoracic presented, the total thoracolumbar count (26 vertebrae) was mostly still conserved, albeit the number of normal lumbar vertebrae was reduced by one. The same trend was also reported in domestic rabbits [27]. This highlighted the highly conserved number of somites developed and determined for pre-sacral vertebrae despite the misexpression of some *Hox* genes in the boundary regions [30,83]. The selection against supernumerary lumbar vertebra could be the higher risk of thoracolumbar fracture due to an excessive degree of flexibility [84].

### 4.3. Other causes of variation

The large within-species samples of this study revealed more variation among individuals and more overlapping variation among species than expected. Unfortunately, individual differences in locomotive ability are not known for these sampled animals; otherwise, this would be an important factor to consider with respect to the observed variation. This leads us to consider the influence of genetic events, particularly genetic drift, in the populations we sampled.

Inheritance of vertebral abnormality (whether it is numerical variance or vertebral anomaly) has been exhaustively tested in domestic *O. cuniculus*—newborn individuals are more likely to inherit abnormality if their parents are abnormal [47,48]. Inbreeding is also found to amplify this effect [28,47,85–87]. Population history differs for our sampled species of leporids: *L. europaeus* and *O. cuniculus* have likely experienced founder effects, and *P. furnessi* has undergone a genetic bottleneck.

The pervasive Australian populations of *L. europaeus* and *O. cuniculus* were established from multiple introductions of small founding colonies into many areas across the country in the mid-nineteenth century [88,89]. As there was no predatory nor competing species to these leporids in Australia in the past, their population exponentially increased, reaching more than millions of reproductively viable animals within less than 50 years [90]. The population size of *O. cuniculus* dramatically fluctuated due to both seasonal drought and pest-control activities, but the effect was less severe on *L. europaeus* populations [90,91]. Inbreeding was very likely in not only the founding population but also the recurring populations as they spread. Our samples of *O. cuniculus* and *L. europaeus* were from multiple localities sampled approximately 150 years after introduction to mitigate population bias, but we cannot discount that these genetic events contribute to the observed variation. An investigation using individuals sourced from their native populations (continental Europe through to Siberia and the south Pacific coast of Russia for *L. europaeus* [66]; central and western Europe for *O. cuniculus* [67]) will provide further insight into the vertebral number variability in these two species.

Japanese endemic populations of *P. furnessi* are confined to Amami-Oshima and Tokunoshima islands, where mammalian predators do not natively exist; their population in Amami-Oshima Island collapsed during the 1980s due to the arrival of alien predators (mongooses and cats) [68,92]. The remaining populations also suffered from low genetic diversity as this species has very low dispersibility, suggested by the lack of gene flow between two nearby populations [93]. Nevertheless, the collapsed population rapidly recovered from approximately 2000 to approximately 22 000 in 20 years [94–97]. Similarly to the *L. europaeus* and *O. cuniculus*, inbreeding was very likely in the recovering populations. But the difference was that the *O. cuniculus* populations had multiple peaks and troughs over a long period of time. This could be a potential factor amplifying the vertebral abnormality in *O. cuniculus*.

Only *L. brachyurus* have not undergone a drastic change in population density in their modern population. They are endemic to Japan, and have been living on Japanese main islands for at least 1.2 million years [98] along with native predators such as foxes, weasels and martens [99]. The predatory pressure acted as a locomotive pressure, constraining the variation as in other fast-running mammals (e.g. [20]). Thus, they may reflect the ‘null’ extent of variation due to locomotive stress in fast-running leporids.

Despite these genetic events, we did not find any CTTV or cervical vertebra with cervical ribs in our dataset. The presence of CTTV or cervical ribs is usually linked with other deleterious traits and has been proposed to be an indicator of a population experiencing inbreeding depression [100–102]. Genetic purging (selective pressure against recessive deleterious alleles) might be a possible cause [102,103]. However, it is more plausible that there are multiple characters that reduce inbreeding depression in leporids, such as the dispersal of juveniles [69,104], or a tendency to breed all year round and have several offspring [91,105].

## 5. Conclusion

This article aimed to test whether the relationship between vertebral numerical variability and running ability known broadly across mammals applies to the finer phylogenetic level. Our results derived from large within-species samples of four leporids did not statistically support this hypothesis. Although the trend was observed, where specialized runners (*Lepus* spp.) showed lower numerical variance and abnormalities than slower species (*O. cuniculus* and *P. furnessi*), it was ambiguous between these two non-specialized runners. This suggests that while the locomotive ability is an evolutionary driver for the general pattern of variability in vertebral numbers, it is not the sole cause; other drivers acting on a shorter time scale, such as the genetic drifting effect, could underlie the observed variation. A novel outcome from our study highlights that vertebral column abnormality is more varied than previously known, with up to 11 different conditions of vertebral abnormality. Since not all variations were observed equally across species, we posit that each abnormal formula could have different impacts on the locomotory performance and an individual’s fitness, thus affecting the heredity of these traits. Overall, further research is needed to understand whether numerical variability in mammals is under stabilizing selection due to fitness, or a developmental response to locomotive constraints (i.e. backbone mobility) in cursorial animals. Our study highlights the importance of comparing patterns of within-species variation with among-species variation to understand how evolutionary diversity comes about (e.g. [106]).

## Data Availability

The vertebral count of each specimen and its respective collection are provided in electronic supplementary table S1 [107]. The digital CT image series of *Lepus europaeus* and *Oryctolagus cuniculus* are available on MorphoSource under project ID 000671799 [108].
